# Impact of liver fibrosis score on prognosis after common therapies for intrahepatic cholangiocarcinoma: a propensity score matching analysis

**DOI:** 10.1186/s12885-020-07051-5

**Published:** 2020-06-15

**Authors:** Jian Xi Zhang, Peipei Li, Zhibin Chen, Huogui Lin, Zhezhen Cai, Weijia Liao, Zirong Pan

**Affiliations:** 1 Department of hepatobiliary surgery, Xiamen Hospital, Beijing University of Chinese Medicine, Xiamen, Fujian China; 2 Department of General Surgery, Xiamen Haicang Hospital, 89 Haiyu Road, Haicang District, Xiamen, Fujian China

**Keywords:** Intrahepatic cholangiocarcinoma, Liver fibrosis, Survival

## Abstract

**Background:**

Liver fibrosis or cirrhosis is associated with the dismal prognosis of hepatocellular carcinoma (HCC), and it might also be involved in intrahepatic cholangiocarcinoma (ICC). The effect of hepatic fibrosis on the survival of ICC patients is still unclear. This study aims to explore whether liver fibrosis impacts the overall survival (OS) and disease-specific survival (DSS) of ICC patients.

**Methods:**

Data of 729 eligible ICC patients receiving different therapies from the Surveillance, Epidemiology, and End Results database (2004–2015) were analyzed. Unmatched, propensity score-matched, and propensity score-weighted cohorts were used to investigate the relationships of different fibrosis scores (low fibrosis score vs. high fibrosis score) and survival. A Cox regression and Kaplan–Meier curves were used to explore the influence of fibrosis score on patients’ survival. Stratified analyses based on treatment modality were conducted to compare the survival difference in ICC patients with different fibrosis scores.

**Results:**

Before matching, the one-, three-, and five-year OS were 50.9, 28.0, and 16.1% in the low fibrosis score group (*n* = 465) and 39.3, 20.1, and 8.0% in the high fibrosis score group (*n* = 264) (*P* < 0.001), respectively. After propensity score matching, the one-, three-, and five-year OS were 45.0, 26.0, and 10.2% in the low fibrosis score group and 36.0, 8.1, and 2.3% in the high fibrosis score group (*P* = 0.008), respectively. The multivariate Cox regression results showed that a high fibrosis score was an independent risk factor of OS. Additionally, patients with high fibrosis scores achieved low DSS after matching (*P* = 0.032). The survival benefits of the low fibrosis score group were consistent across treatment cohorts.

**Conclusions:**

High fibrosis scores were associated with poor clinical outcomes of ICC patients receiving different common therapies.

## Background

Intrahepatic cholangiocarcinoma (ICC) is a relatively rare type of primary hepatic malignancy with high invasive features, and its incidence is second to that of hepatocellular carcinoma (HCC) [1]. However, the morbidity and mortality of ICC have been increasing globally during the past decades. As a result of late diagnosis, ICC frequently presents with large and/or multiple tumors [2, 3]. Currently, the major treatment modalities consist of surgical resection, regional therapy, chemotherapy, and liver transplantation [4], but the long-term prognosis of ICC remains disappointing because of the high recurrence rate [5].

Several risk factors have been reported to be associated with the occurrence and development of ICC, and they include hepatolithiasis, hepatitis B and C virus infection, liver cirrhosis, and primary sclerosing cholangitis [6, 7]. Fibrosis (Fb), a liver tissue scar reaction involved in various types of chronic liver damage, is a complicated process with multiple steps ranging from chronic liver disease to cirrhosis [8]. In fact, more than 80% of HCC cases are known to have developed in the setting of fibrosis or cirrhosis, with chronic inflammation, tissue regeneration, and other molecular events resulting in the production of reactive oxygen species, chromosomal mutations, and, eventually, the malignant transformation of proliferating hepatocytes [9].

A limited number of studies have explored the relationships between liver fibrosis and the clinicopathological characteristics of ICC. Recent evidence suggests that cancer-associated fibroblasts (CAFs) and fibrosis in the tumor microenvironment promote the development and progression of cholangiocarcinoma (CCA) through multiple mechanisms involving multicellular signaling networks [10]. Therefore, in the current study, we aim to investigate the impact of fibrosis on the overall survival (OS, measured from the date of diagnosis to the date of death) and disease-specific survival (DSS, defined as the interval from the date of diagnosis to the date of cancer-specific death [11]) of ICC patients from the Surveillance, Epidemiology, and End Results (SEER) database (http://www.seer.cancer.gov) of the National Cancer Institute. This program has been published routinely and has archived 21 population-based cancer registries. It has also been deemed applicable to cancer-based epidemiology and survival analyses because of its comprehensive information coverage, which includes demographics, primary tumor site, tumor morphology, stage at diagnosis, main therapeutic course, follow-up records, etc.

## Methods

### Data source and study population

**Fig. 1:**
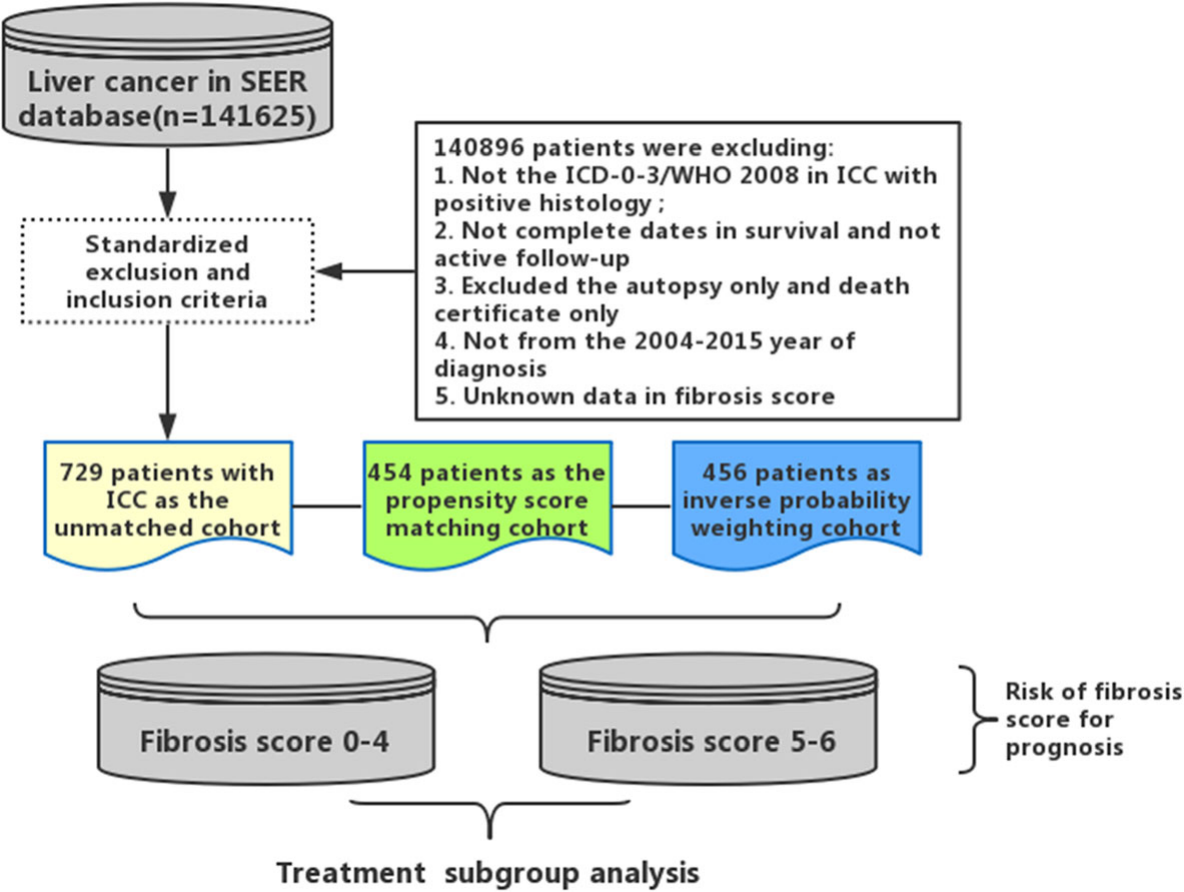
Flowchart displaying the selection procedure of ICC cases in the SEER database

The database used in our study was the incidence–SEER 18 Regs Custom data (with additional treatment fields), Nov2018 Sub (1975–2016 varying). A total of 729 ICC patients were identified at clinical stages I–IV from a population of 141,625 candidates diagnosed with primary liver cancer between 2004 and 2015. Of the identified patients, 63.8% of them (n = 465) were assigned to the low fibrosis score (low-Fb score) group (Fb score of 0–4), and the remaining ones (n = 264, 36.2%) were assigned to the high fibrosis score (high-Fb score) group (Fb score of 5–6). The flowchart of this study is presented in Fig. 1, and details are provided in Supplementary file 1.

### Parameters

Variables from the patient, tumor, and treatment levels were adopted in the statistical analysis. The patient-level parameters included age at diagnosis, sex, race, and marital status. The tumor-level variables included tumor number, tumor size, year of diagnosis (2004–2009 vs. 2010–2015), alpha-fetoprotein (AFP, cutoff value of 15 ng/mL), lymph node status, metastasis of diagnosis, tumor pathological grade, tumor node metastasis (TNM) stage, and Ishak Fb score. The treatment-level variables mainly included the surgery, radiation, and chemotherapy records of ICC patients. A full description of the variables is available in the SEER Data Management System User Manual (https://seer.cancer.gov/tools/codingmanuals/index.html) and CS Coding Instructions v0205.

### Statistical analysis

Continuous variables were demonstrated as means with standard deviations (SD) and were compared using the student’s t-test or Mann–Whitney U test. Categorical variables, presented as frequencies and percentages (%), were compared using the chi-square test or Fisher’s exact test.

To balance the differences between the two groups, we constructed a propensity score model by using the variables of the entire logistic regression model for patients with high-Fb scores. The candidate variables included all variables significantly associated with high-Fb scores, as determined via a univariate analysis with a threshold of *P* < 0.2. Then, propensity score matching (PSM) and inverse probability of weighting (IPW) methods were adopted to reduce the standard mean differences (SMDs) of the covariates. In PSM, individuals with low-Fb scores were matched to those with high-Fb scores by using a matching ratio of approximately 1:1, with the closest estimated propensity score values limited within 0.1 of the SD in the study population [12]. All patients with high- and low-Fb scores were weighted by the propensity score model via the IPW method [13, 14]. Two matched cohorts (PSM cohort and IPW cohort) were generated to validate the associations of the Fb scores with the clinical outcomes of ICC patients. The Kaplan–Meier (KM) curves for the OS and compete risk survival analysis were derived to visualize the comparison between the high- and low-Fb scores. Cox regression analysis was performed to explore the risk factors influencing patient survival. Additionally, the treatment benefits for the ICC patients with high- and low-Fb scores were further explored and confirmed in the matched cohorts via stratified analyses based on treatment modalities. All the statistical analyses were performed in R (R Foundation for Statistical Computing, Vienna, Austria, Version 3.6.0), and the R packages of “forestplot,” “glm,” “ggolot2,” “matching,” “survey,” and “cmprsk” were used. Two-tailed P < 0.05 was considered to indicate statistical significance.

## Results

### Baseline characteristics

**Fig. 2:**
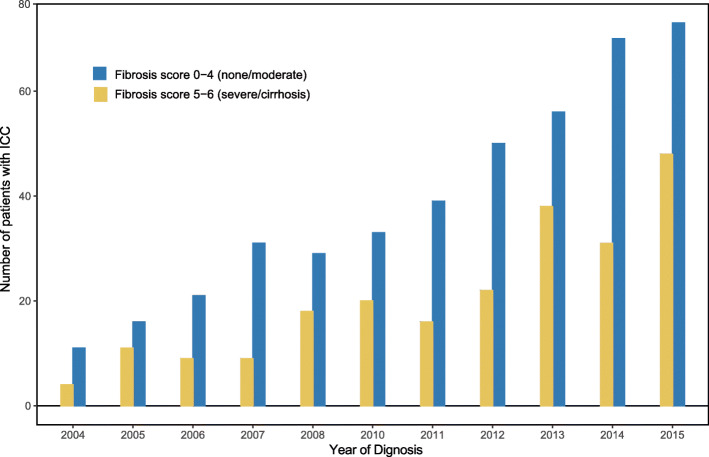
Number of patients with low-Fb scores versus high-Fb scores over time in the National Cancer Center Database, 2004–2015

According to the inclusion and exclusion criteria, 729 eligible ICC patients from the SEER database (2004–2015) were analyzed (Fig. 1). The baseline clinicopathological characteristics of the ICC patients in the high- and low-Fb score groups are listed in Table 1. As shown in Fig. 2, despite the rising incidence, the proportion of patients with high-Fb scores did not significantly change over time.

**Table 1:** Demographic and clinical characteristics of ICC patients with different fibrosis score

Variables	Low-fibrosis score group	High-fibrosis score group	*P* *Value*
No. of patient, n (%)	465 (63.9)	264 (36.1)	
Marital status, n (%)			0.188
Married	287 (61.7)	146 (55.3)	
Single	157 (33.8)	107 (40.5)	
Unknown	21 (4.5)	11 (4.2)	
Age (mean (sd))	65.94 (11.5)	64.21 (9.9)	0.040
Age, ≤60, years, n (%)	143 (30.8)	92 (34.8)	0.292
Sex, male, n (%)	218 (46.9)	174 (65.9)	< 0.001
Year of diagnosis, 2010–2015, n (%)	321 (69.0)	193 (73.1)	0.282
Ethnicity, n (%)			0.053
Black	31 (6.7)	21 (8.0)	
White	350 (75.3)	213 (80.7)	
Other	84 (18.1)	30 (11.4)	
AFP, ng/ml, n (%)			< 0.001
≤ 15	219 (47.1)	113 (42.8)	
> 15	63 (13.5)	79 (29.9)	
Unknown	183 (39.4)	72 (27.3)	
Number of tumors, single, n (%)	338 (72.7)	208 (78.8)	0.082
Tumor size, cm, n (%)			0.013
> 3	323 (69.5)	160 (60.6)	
≤ 3	63 (13.5)	57 (21.6)	
Unknown	79 (17.0)	47 (17.8)	
Lymph nodes metastasis, n (%)			0.499
No	341 (73.3)	190 (72.0)	
Yes	112 (24.1)	63 (23.9)	
Unknown	12 (2.6)	11 (4.2)	
Distance metastasis, n (%)			0.013
No	361 (77.6)	181 (68.6)	
Yes	98 (21.1)	81 (30.7)	
Unknown	6 (1.3)	2 (0.8)	
6th AJCC TNM stage, n (%)			0.008
I	135 (29.0)	84 (31.8)	
II	54 (11.6)	24 (9.1)	
III	144 (31.0)	55 (20.8)	
IV	102 (21.9)	83 (31.4)	
Unstaged	30 (6.5)	18 (6.8)	
Pathological grade, n (%)			0.009
Grade I	35 (7.5)	12 (4.5)	
Grade II	166 (35.7)	74 (28.0)	
Grade III	106 (22.8)	57 (21.6)	
Grade IV	5 (1.1)	1 (0.4)	
Unstaged	153 (32.9)	120 (45.5)	
Surgery record, yes, n (%)	241 (51.8)	97 (36.7)	< 0.001
Radiation record, yes, n (%)	74 (15.9)	35 (13.3)	0.391
Chemotherapy record, yes, n (%)	215 (46.2)	109 (41.3)	0.224
**Outcomes**			
Survival month (mean (sd))	22.43 (24.74)	16.67 (21.95)	0.002
Vital status, dead, n (%)	342 (73.5)	212 (80.3)	0.050
Compete risk, n (%)			0.103
Alive	123 (26.5)	52 (19.7)	
Dead with other reasons	23 (4.9)	17 (6.4)	
Dead with cancer	319 (68.6)	195 (73.9)	

Abbreviation: *AFP* serum alpha fetoprotein, *AJCC* American Joint Committee on cancer

### Overall survival analysis and competing risk survival analysis before matching

**Fig. 3:**
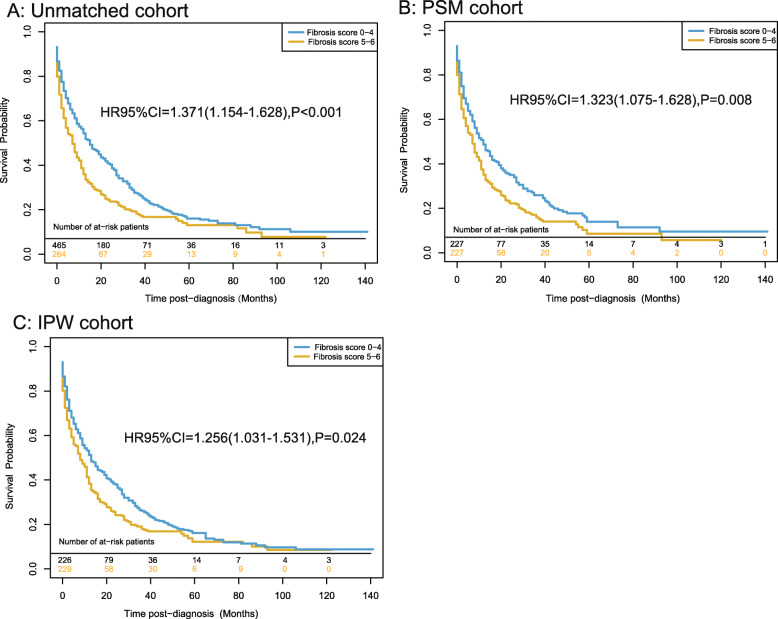
Overall survival rates in (**a**) the unmatched, (**b**) the propensity score-matched, and (**c**) the inverse probability of treatment weight-adjusted analysis of ICC patients

**Fig. 4:**
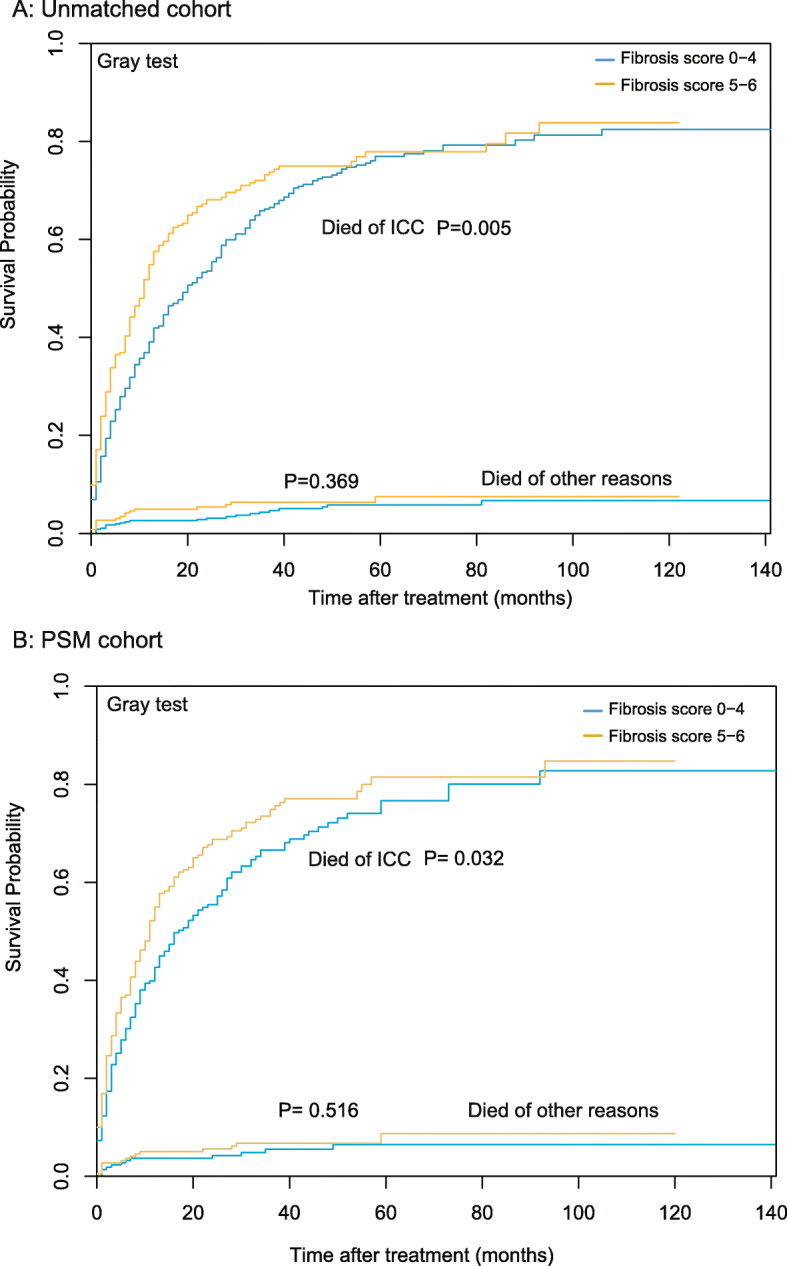
Disease-specific survival in (**a**) the unmatched and (**b**) the propensity score-matched analysis of ICC patients

The median survival times in the low- and high-Fb score groups were 22.43 and 16.67 months, respectively. The OS analysis (Fig. 3a) showed that a high-Fb score was a significant risk factor of OS in ICC patients (HR 95%CI = 1.371 [1.154–1.628], *P* < 0.001). Before matching, the one-, three-, and five-year OS rates were 50.9, 28.0, and 16.1% in the low-Fb score group and 39.3, 20.1, and 8.0% in the high-Fb score group (*P* < 0.001), respectively. Table 2 shows the results of the univariate and multivariate Cox regression analyses in the unmatched cohort. We found that a high-Fb score was an independent risk factor of OS in ICC patients (HR 95%CI = 1.282 [1.067–1.541], *P* = 0.008). In addition to the Fb score, we identified being male, presence of multiple tumors, tumor size > 3 cm, presence of distance metastasis, and advanced stage as risk factors of OS. Meanwhile, surgery treatment and chemotherapy were identified as protective factors of OS in ICC patients. In the competing risk survival analysis, the unadjusted P values of the Fb score for the death circumstance of ICC and other reasons were 0.005 and 0.369 in the primary cohort (Fig. 4a).

**Table 2:** The univariate and multivariate Cox analysis of overall survival in the unmatched cohort

	Univariate analysis			Multivariate analysis		
Characteristics	HR	95%CI	*P value*	HR	95%CI	*P value*
Sex, Male vs. Female	1.239	1.047–1.466	0.013	1.386	1.157–1.661	< 0.001
Age years, ≤60 vs. > 60	0.838	0.701–1.003	0.054			
Marital status						
Married	ref					
Single	1.211	1.017–1.441	0.032			
Unknown	1.175	0.788–1.752	0.430			
Year of diagnosis, 2010–2015 vs. Elarly	0.838	0.702–1.001	0.052			
Ethnicity						
Black	ref					
Other	0.760	0.52–1.112	0.157			
White	0.917	0.66–1.273	0.605			
AFP, ng/ml	ref					
> 15 vs. ≤15	1.140	0.906–1.434	0.263			
Unknown vs. ≤15	1.134	0.925–1.391	0.225			
Fibrosis score, 5–6 vs. 0–4	1.371	1.154–1.628	< 0.001	1.282	1.067–1.541	0.008
Number of tumors, Multi vs. Single	1.385	1.138–1.685	0.001	1.548	1.258–1.904	< 0.001
Tumor size, cm						
3 cm	ref					
≤ 3 cm	0.475	0.364–0.62	< 0.001	0.517	0.39–0.684	< 0.001
Unknown	2.029	1.647–2.499	< 0.001			
Lymph nodes metastasis, No	ref					
Yes	1.738	1.434–2.107	< 0.001			
Unknown	2.240	1.427–3.516	< 0.001			
Distance metastasis, No	ref					
Yes	3.605	2.976–4.368	< 0.001	2.376	1.112–5.078	0.026
Unknown	5.010	2.48–10.118	< 0.001	2.792	1.035–7.533	0.043
6th AJCC TNM stage						
I	ref					
II	1.471	1.065–2.032	0.019	1.934	1.39–2.692	< 0.001
III	2.291	1.806–2.907	< 0.001	2.495	1.776–3.506	< 0.001
IV	5.402	4.245–6.875	< 0.001	1.750	0.695–4.403	0.235
Unstaged	3.974	2.811–5.618	< 0.001	4.144	2.481–6.92	< 0.001
Pathological grade						
I	ref					
II	0.940	0.64–1.38	0.751	0.669	0.452–0.991	0.045
III	1.545	1.043–2.288	0.030	0.988	0.659–1.481	0.954
IV	1.206	0.424–3.4	0.731	0.718	0.247–2.082	0.542
Unstaged	2.026	1.391–2.951	< 0.001	1.235	0.831–1.834	0.297
Surgery record, Yes vs. No	0.221	0.184–0.266	< 0.001	0.305	0.242–0.383	< 0.001
Radiation record, Yes vs. No	0.874	0.692–1.102	0.255			
Chemotherapy record, Yes vs. No	0.830	0.702–0.983	0.031	0.490	0.407–0.589	< 0.001

Abbreviation: *HR* Hazard ratio, *CI* confidence interval, *AFP* serum alpha fetoprotein, *AJCC* American Joint Committee on cancer

### Factors associated with high-Fb scores in ICC patients

A propensity score model was constructed on the basis of the multivariate logistic regression analysis (Table 3). Compared with the patients in the low-Fb score group, those in the high-Fb score group were mostly single and male and were more likely to have higher AFP levels, small tumor size (≤3 cm), and multiple tumors. The 6th AJCC stage III was more inclined to occur in patients with high-Fb scores than in their counterparts.

**Table 3:** Propensity modeling of high fibrosis score (severe/cirrhosis) patients

Variables	OR	95%CI	*P value*
Age, years			
> 60	Reference		
≤ 60	0.999	0.929–1.074	0.987
Sex			
Female	Reference		
Male	1.177	1.099–1.261	**< 0.001**
Marital status			
Married	Reference		
Single	1.082	1.007–1.164	**0.032**
Unknown	1.057	0.894–1.248	0.523
Ethnicity			
Black	Reference		
White	0.978	0.854–1.120	0.749
Other	0.887	0.760–1.035	0.129
AFP, ng/ml			
≤ 15	Reference		
> 15	1.217	1.109–1.334	**< 0.001**
Unknown	0.959	0.888–1.035	0.286
Tumor size, cm			
> 3 cm	Reference		
≤ 3 cm	1.165	1.059–1.283	**0.002**
Unknown	0.975	0.881–1.080	0.635
Number of tumors			
Single	Reference		
Multiple	1.083	1.002–1.172	**0.045**
Distance metastasis			
No	Reference		
Yes	1.091	0.816–1.458	0.559
Unknown	0.908	0.639–1.289	0.587
6th AJCC TNM stage			
I	Reference		
II	0.92	0.817–1.037	0.174
III	0.901	0.824–0.986	**0.025**
IV	0.94	0.700–1.261	0.68
Unstaged	1.018	0.862–1.203	0.836
Pathological grade			
I	Reference		
II	1.038	0.899–1.197	0.619
III	1.097	0.944–1.276	0.226
IV	0.971	0.656–1.438	0.886
Unstaged	1.16	1.003–1.340	**0.047**

Abbreviation: *OR* odd ratio, *CI* confidence interval, *AFP* serum alpha fetoprotein, *AJCC* American Joint Committee on cancer

### Balance between high- and low-Fb score groups

According to the propensity score model (Table 3), PSM, excluding the prognosis indexes (vital status and survival time), achieved an adequate balance between the high- and low-Fb score groups. Notably, we found a decrease in SMDs between the two groups in the IPW analysis but not in PSM (Supplemental Table 1 in Supplemental file 1). After PSM and IPW analyses, the variables between the two groups were significantly balanced because all the available *P* values were > 0.05.

### Overall survival analysis and competing risk survival analysis after matching

After matching, the KM curves (Fig. 3b and c) showed that a high-Fb score was a significant risk factor of OS in the matched cohort (HR 95%CI = 1.323 [1.075–1.628], *P* = 0.008). IPW analysis revealed similar results (HR 95%CI = 1.256 [1.031–1.531], *P* = 0.024). After PSM, the one-, three-, and five-year OS rates were 45.0, 26.0, and 10.2% in the low-Fb score group and 36.0, 8.1, and 2.3% in the high-Fb score group (*P* = 0.008), respectively. In the competing risk survival analysis, the propensity score-adjusted P values were 0.032 and 0.516 in the matching cohort after PS matching, (Fig. 4b).

### Survival benefits of low-Fb scores in patients with surgical treatment

**Fig. 5:**
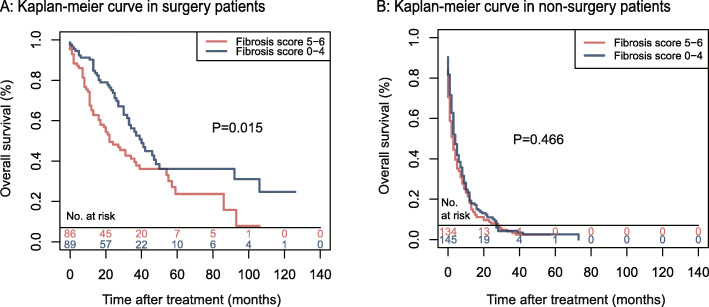
Overall survival in (**a**) the surgery recorded group and (**b**) the nonsurgery recorded group of matched cohort

Figure 5 shows the comparison results of the OS rates of patients who received surgery with different Fb scores after PSM. Compared with the patients in the low-Fb score group, those in the high-Fb score group had poorer OS rates, with the P values of the log-rank test between the two groups being 0.015 (Fig. 5a). In the comparison of the OS rates of patients without surgery records, different results were observed between the two groups (*P* = 0.466) (Fig. 5b). The subgroup survival analyses outcomes between the two groups of patients with and without the other two therapies are illustrated in Supplemental Figure 1. A subgroup analysis of the prognosis of ICC patients with different therapies was conducted. Supplemental Figure 2 shows that in the surgery group, the patients’ survival outcomes were not influenced by the treatment they received (i.e., chemotherapy and radiotherapy treatments) (*P* = 0.96 and 0.79). By contrast, chemotherapy and radiotherapy were significantly related to the prognosis of ICC patients in the none-surgery group (*P* < 0.001).

## Discussion

HCC is significantly correlated with liver fibrosis, and 80–90% of patients developed HCC with fibrotic or cirrhotic liver settings [9]. However, due to the low incidence of ICC, few studies have focused on the role of liver fibrosis in the pathogenesis and mechanism of ICC. Recently, several researchers have found that CCAs usually occur in nonfibrotic livers but that they subsequently show a strong pro-connective tissue proliferation response similar to pancreatic cancer [15]. Sirica AE et al. contended that CCA is characterized by a large number of desmoplastic reactions that do not depend on surrounding tissue fibrosis and can impact the values of Fb scores or other related indicators [16]. The desmoplastic and hypovascularized nature of CCA is known to contribute to poor prognosis and therapy resistance [10].

In our study, we found that a high-Fb score was significantly related to poor OS and DSS after PSM. The poor OS of patients with high-Fb scores was consistent after IPW analyses. Using the stratification analysis, we found that a high liver Fb score was significant in the poor prognostic outcomes of patients with records of common clinical treatments (surgery, radiation, and systemic chemotherapy). In addition, patients characterized as male and single and having AFP > 15 ng/mL, tumor size ≤3 cm, multiple tumors, and stage III of the 6th AJCC stage were likely to have high-Fb scores.

At present, only a few studies have formally assessed the impact of Fb score on the prognosis of ICC patients after treatment. The current study is the first to explore the relationship between Fb score and ICC prognosis using the national SEER database. The results of our study demonstrated that relative to low-Fb scores, high-Fb scores impaired patients’ survival outcomes, which were independent of the treatment methods. Moreover, ICC patients with low-Fb scores could obtain more survival benefits than those with high-Fb scores. Propensity scores were generated on the basis of a large number of factors that could affect treatment allocation, likely attenuating the possibility of allocation bias. We also attempted to control for an unmeasured confounder, that is, the presence of cirrhosis or advanced fibrosis, by using subgroup analyses.

Numerous researchers have reported the impact of fibrosis on HCC survival. In 2018, Sivesh K used the national SEER database and found that HCC patients with Fb scores of 0–4 had better survival rates than those with high-Fb scores after liver resection [17]. Hui Liu et al. found that Fb score is an independent prognostic factor for OS but not for DSS [18]. In 2014, Taiwan researchers found that severe liver fibrosis exerts a negative impact on OS and DSS in small HCC patients and that fibrosis is an independent predictor of tumor recurrence among patients who undergo hepatectomy [19]. At present, no study has explored the relationship between the degree of liver fibrosis and prognosis in ICC. In our work, we included a large number of ICC patients regardless of the treatments they received. Our results are consistent with the aforementioned studies in HCC.

Researchers have found that CAFs are complex in terms of their contribution to the development and growth of CCA. Fibroblasts and the extracellular matrix (ECM) may play important tumor-promoting and tumor-restricting roles [10]. Reports have shown that a high number of myofibroblasts or high expression of ECM proteins, such as periostin, is associated with a significantly low survival rate in CCA patients [20–22]. In the tumor microenvironment, CAFs produce and secrete a large amount of ECM proteins, and clinical evidence proves that stromal cells play an important role in tumor development. The aforementioned research might explain why patients with high-Fb scores are prone to having nonideal survival outcomes.

The mechanisms of how myofibroblasts and fibrosis affect the development and progression of CCA are intricate. Current evidence shows that CAFs promote the development and progression of CCA through a variety of mechanisms, which are likely to work together in complex multicellular signal networks. For example, a research found that tumor cells in ICC might produce a factor responsible for activating myofibroblasts and that the capacity of activation varies in each ICC patient [21]. Claperon A et al. found that the cotransplantation of CCA tumor cells with human liver myofibroblasts increases tumor incidence, size, and metastatic dissemination in vivo; this effect can be inhibited by the EGFR tyrosine kinase inhibitor gefitinib [23]. Moreover, coculture experiments demonstrate EGFR activation in tumor cells by myofibroblast-derived EGF, resulting in enhanced migratory and invasive properties in vitro [23]. Fingas CD et al. found that myofibroblast-derived PDGF-BB promotes Hedgehog signaling-dependent survival signals in CCA cells [24]. Aberrant Hedgehog signaling between stromal myofibroblastic cells and CCA cells is a critical modulator of intrahepatic CCA progression and therapy resistance. The main mechanism by which CAFs promote the progression of CCA is to support the proliferation and survival of tumor cells through the expression of cytokines and growth factors, such as periostin, thrombospondin-1, SDF-1, MMP2, MMP9, and IL-1β. Another mechanism is that cancer-associated fibroblasts modulate inflammation and immune responses in CCA [10]. SDF-1 is the best characterized CAF-derived inflammatory mediator in CCA.

This study has several potential limitations. First, the SEER database lacks information on the fibrosis background of ICC patients. The SEER database also groups together patients with Fb scores of 0–4 and 5–6; hence, we could not perform our analysis according to different Fbs. Second, the pathological information of tumors in the SEER database is not openly available. Therefore, accurate TNM staging (the SEER database only contains the 6th AJCC stage) was not possible in this study. Third, the data on postoperative complications in the SEER database were limited, and a few patients received more than one treatment. These issues may affect the observation of the results of the impact of fibrosis on prognosis. Finally, SEER information comes from different registries. Hence, mistakes pertaining to the accuracy of the data are inevitable because no specialized staff is responsible for checking the data completely.

## Conclusions

In liver, fibrosis is related to the survival outcome of ICC patients. It can be regarded as a predictor of prognosis for ICC patients who have received common therapies. Patients with low-Fb scores could benefit greatly from ICC treatments.

## Supplementary information

**Additional file 1:**

**Additional file 2: Supplemental Figure 1.** Subgroup survival analyses for the prognosis of ICC patients with fibrosis scores.

**Additional file 3: Supplemental Figure 2.** Subgroup analysis for the prognosis of ICC patients receiving different therapies.

## Data Availability

The datasets generated and/or analysed during the current study are available in the Surveillance, Epidemiology, and End Results (SEER) database (http://www.seer.cancer.gov) of the National Cancer Institute.

## Abbreviations

HCC: Hepatocellular carcinoma
ICC: Intrahepatic cholangiocarcinoma
OS: Overall survival
DSS: Disease-specific survival
Fb: Fibrosis
CAF: Cancer-associated fibroblasts
CCA: Cholangiocarcinoma
SEER: Surveillance, Epidemiology, and End Results
AFP: Alpha-fetoprotein
TNM: Tumor node metastasis
SD: Standard deviations
PSM: Propensity score matching
IPW: Inverse probability of weighting
SMD: Standard mean difference
KM: Kaplan–Meier
HR: Hazard ratio
AJCC: American Joint Committee on Cancer
ECM: Extracellular matrix
EGFR: Epidermal Growth Factor Receptor
PDGF-BB: Platelet-derived growth factors BB
SDF-1: Stromal cell-derived factor-1
MMP2: Matrix metalloproteinase 2
MMP9: Matrix metalloproteinase 9
IL-1: Interleukin-1

## References

[1] Wang M, Gao Y, Feng H, Warner E, An M, Jia J, Chen S, Fang M, Ji J, Gu X (2018). A nomogram incorporating six easily obtained parameters to discriminate intrahepatic cholangiocarcinoma and hepatocellular carcinoma. Cancer Med.

[2] Adachi T, Eguchi S (2014). Lymph node dissection for intrahepatic cholangiocarcinoma: a critical review of the literature to date. J Hepatobiliary Pancreat Sci.

[3] Wang K, Zhang H, Xia Y, Liu J, Shen F (2017). Surgical options for intrahepatic cholangiocarcinoma. Hepatobiliary Surg Nutr.

[4] Rahnemai-Azar AA, Weisbrod AB, Dillhoff M, Schmidt C, Pawlik TM (2017). Intrahepatic cholangiocarcinoma: current management and emerging therapies. Expert Rev Gastroenterol Hepatol.

[5] Hu LS, Weiss M, Popescu I, Marques HP, Aldrighetti L, Maithel SK, Pulitano C, Bauer TW, Shen F, Poultsides GA (2019). Impact of microvascular invasion on clinical outcomes after curative-intent resection for intrahepatic cholangiocarcinoma. J Surg Oncol.

[6] Razumilava N, Gores GJ (2014). Cholangiocarcinoma. Lancet.

[7] Siegel RL, Miller KD, Jemal A (2017). Cancer statistics, 2017. CA Cancer J Clin.

[8] Friedman SL (2010). Evolving challenges in hepatic fibrosis. Nat Rev Gastroenterol Hepatol.

[9] El-Serag HB (2011). Hepatocellular carcinoma. N Engl J Med.

[10] Affo S, Yu LX, Schwabe RF (2017). The role of Cancer-associated fibroblasts and fibrosis in liver Cancer. Annu Rev Pathol.

[11] Rosthoj S, Andersen PK, Abildstrom SZ (2004). SAS macros for estimation of the cumulative incidence functions based on a cox regression model for competing risks survival data. Comput Methods Prog Biomed.

[12] Austin PC (2011). An introduction to propensity score methods for reducing the effects of confounding in observational studies. Multivariate Behav Res.

[13] Austin PC, Stuart EA (2015). Moving towards best practice when using inverse probability of treatment weighting (IPTW) using the propensity score to estimate causal treatment effects in observational studies. Stat Med.

[14] Liang L, Tom G (2013). A weighting analogue to pair matching in propensity score analysis. Int J Biostat.

[15] Sirica AE (2011). The role of cancer-associated myofibroblasts in intrahepatic cholangiocarcinoma. Nat Rev Gastroenterol Hepatol.

[16] Sirica AE, Gores GJ (2014). Desmoplastic stroma and cholangiocarcinoma: clinical implications and therapeutic targeting. Hepatology.

[17] Kamarajah SK (2018). Fibrosis score impacts survival following resection for hepatocellular carcinoma (HCC): a surveillance, end results and epidemiology (SEER) database analysis. Asian J Surg.

[18] Liu H, Cen D, Yu Y, Wang Y, Liang X, Lin H, Cai X (2018). Does fibrosis have an impact on survival of patients with hepatocellular carcinoma: evidence from the SEER database?. BMC Cancer.

[19] Ko CJ, Lin PY, Lin KH, Lin CC, Chen YL (2014). Presence of fibrosis is predictive of postoperative survival in patients with small hepatocellular carcinoma. Hepatogastroenterology.

[20] Chuaysri C, Thuwajit P, Paupairoj A, Chau-In S, Suthiphongchai T, Thuwajit C (2009). Alpha-smooth muscle actin-positive fibroblasts promote biliary cell proliferation and correlate with poor survival in cholangiocarcinoma. Oncol Rep.

[21] Okabe H, Beppu T, Hayashi H, Horino K, Masuda T, Komori H, Ishikawa S, Watanabe M, Takamori H, Iyama K (2009). Hepatic stellate cells may relate to progression of intrahepatic cholangiocarcinoma. Ann Surg Oncol.

[22] Utispan K, Thuwajit P, Abiko Y, Charngkaew K, Paupairoj A, Chau-in S, Thuwajit C (2010). Gene expression profiling of cholangiocarcinoma-derived fibroblast reveals alterations related to tumor progression and indicates periostin as a poor prognostic marker. Mol Cancer.

[23] Claperon A, Mergey M, Aoudjehane L, Ho-Bouldoires TH, Wendum D, Prignon A, Merabtene F, Firrincieli D, Desbois-Mouthon C, Scatton O (2013). Hepatic myofibroblasts promote the progression of human cholangiocarcinoma through activation of epidermal growth factor receptor. Hepatology.

[24] Fingas CD, Bronk SF, Werneburg NW, Mott JL, Guicciardi ME, Cazanave SC, Mertens JC, Sirica AE, Gores GJ (2011). Myofibroblast-derived PDGF-BB promotes hedgehog survival signaling in cholangiocarcinoma cells. Hepatology.

