# Supplementary material for: Impact of liver fibrosis score on prognosis after common therapies for intrahepatic cholangiocarcinoma: a propensity score matching analysis
Source: BMC Cancer. 2020 Jun 15;20:556. doi: 10.1186/s12885-020-07051-5 (PMC7296657; doi:10.1186/s12885-020-07051-5)
Supplement: Supplementary file 1 — Additional file 1: [file 12885_2020_7051_MOESM1_ESM.docx]

**The filter principles for ICC patients who had the complete data in fibrosis score included the following items**

1、Site recode ICD-0-3/WHO 2008: liver and intrahepatic bile duct **(n=141625)**

2、Site and morphology diagnostic confirmation: positive histology **(n=81309)**

3、Cause of Death and Follow-up, Survival month’s flag/type of follow-up expected: complete dates are available and there are more than 0 days of survival/ Active follow-up. **(n=73146)**

4、Other type of reporting source: Autopsy only, death certificate only. (**n=73112)**

5、Year of diagnosis (2004-2015) **(n=47964)**

6、ICD-O-3 Hist/behave: Cholangiocarcinoma and Histologic Type ICD-O-3: 8160 **(n=6633)**

7、Excluded the unknown data **in the** Site-Specific Factor 2 – Fibrosis Score (Liver, Intrahepatic Bile Ducts) **(n=5904)**

8、Last analysis data for Cholangiocarcinoma **(n=729)**

**Supplementary table1. comparison of baseline variables between fibrosis score (0-4) and fibrosis score (5-6) groups in the matched dataset with standard difference of different variables from the original data, PSM data and the IPW data**

|  | **SMD** | | | **PS matched cohort** | | | **IPW cohort** | | |
| --- | --- | --- | --- | --- | --- | --- | --- | --- | --- |
| **Variables** | **Unadjusted** | **PSM** | **IPW** | **Fibrosis Score (0-4)** | **Fibrosis Score (5-6)** | ***P***  ***Value*** | **Fibrosis Score (0-4)** | **Fibrosis Score (5-6)** | ***P***  ***Value*** |
| No. of patient, n (%) |  |  |  | 227 | 227 |  | 226.52 | 229.14 |  |
| Marital status, n (%) | 0.141 | 0.041 | 0.05 |  |  | 0.911 |  |  | 0.991 |
| Married |  |  |  | 127 (56.8) | 125 (55.0) |  | 124.0 (54.7) | 125.8 (54.9) |  |
| Single |  |  |  | 90 (38.8) | 93 (41.0) |  | 94.7 (41.8) | 94.3 (41.1) |  |
| Unknown |  |  |  | 10 (4.4) | 9 (4.0) |  | 7.9 (3.5) | 9.1 (4.0) |  |
| Age (mean (sd)) | 0.161 | 0.088 | 0.08 | 65.06 (10.95) | 64.66 (10.23) | 0.687 | 65.31 (11.25) | 64.47 (10.08) | 0.341 |
| Age, ≤60, years, n (%) | 0.087 | 0.019 | <0.001 | 83 (36.6) | 77 (33.9) | 0.623 | 77.8 (34.3) | 77.6 (33.9) | 0.913 |
| Sex, male (%) | 0.391 | 0.019 | 0.04 | 139 (61.2) | 139 (61.2) | 1.000 | 139.8 (61.7) | 141.9 (61.9) | 0.963 |
| Year of diagnosis, 2010-2015, n (%) | 0.090 | 0.021 | 0.04 | 167 (73.6) | 165 (72.7) | 0.916 | 165.4 (73.0) | 167.9 (73.3) | 0.954 |
| Ethnicity, n (%) | 0.192 | 0.078 | 0.04 |  |  | 0.867 |  |  | 0.970 |
| Black |  |  |  | 16 (7.0) | 19 (8.4) |  | 17.6 (7.8) | 19.0 (8.3) |  |
| White |  |  |  | 181 (79.7) | 179 (78.9) |  | 179.9 (79.4) | 181.3 (79.1) |  |
| Other |  |  |  | 30 (13.2) | 29 (12.8) |  | 28.9 (12.8) | 28.8 (12.6) |  |
| AFP, ng/ml, n (%) | 0.423 | 0.012 | 0.08 |  |  | 0.859 |  |  | 0.961 |
| ≤15 |  |  |  | 99 (43.6) | 104 (45.8) |  | 103.1 (45.5) | 106.7 (46.6) |  |
| >15 |  |  |  | 56 (24.7) | 56 (24.7) |  | 52.6 (23.2) | 53.1 (23.2) |  |
| Unknown |  |  |  | 72 (31.7) | 67 (29.5) |  | 70.8 (31.2) | 69.4 (30.3) |  |
| Number of tumors, single, n (%) | 0.143 | 0.054 | 0.01 | 178 (78.4) | 176 (77.5) | 0.910 | 176.1 (77.8) | 177.6 (77.5) | 0.943 |
| Tumor size, cm, n (%) | 0.223 | 0.082 | 0.01 |  |  | 0.593 |  |  | 0.982 |
| >3 |  |  |  | 146 (64.3) | 145 (63.9) |  | 146.2 (64.6) | 146.4 (63.9) |  |
| ≤3 |  |  |  | 43 (18.9) | 37 (16.3) |  | 39.9 (17.6) | 41.5 (18.1) |  |
| Unknown |  |  |  | 38 (16.7) | 45 (19.8) |  | 40.4 (17.8) | 41.2 (18.0) |  |
| Lymph nodes metastasis, n (%) | 0.088 | 0.045 | 0.12 |  |  | 0.595 |  |  | 0.388 |
| No |  |  |  | 165 (72.7) | 165 (72.7) |  | 168.0 (74.1) | 165.2 (72.1) |  |
| Yes |  |  |  | 55 (24.2) | 51 (22.5) |  | 52.9 (23.4) | 53.4 (23.3) |  |
| Unknown |  |  |  | 7 (3.1) | 11 (4.8) |  | 5.6 (2.5) | 10.5 (4.6) |  |
| Distance metastasis, n (%) | 0.224 | 0.058 | 0.03 |  |  | 0.901 |  |  | 0.982 |
| No |  |  |  | 158 (69.6) | 158 (69.6) |  | 157.6 (69.6) | 161.1 (70.3) |  |
| Yes |  |  |  | 66 (29.1) | 67 (29.5) |  | 67.0 (29.6) | 66.0 (28.8) |  |
| Unknown |  |  |  | 3 (1.3) | 2 (0.9) |  | 1.9 (0.8) | 2.0 (0.9) |  |
| 6^th^ AJCC TNM stage, n (%) | 0.290 | 0.144 | 0.02 |  |  | 0.997 |  |  | 1.000 |
| I |  |  |  | 68 (30.0) | 67 (29.5) |  | 70.1 (30.9) | 72.2 (31.5) |  |
| II |  |  |  | 23 (10.1) | 22 (9.7) |  | 21.3 (9.4) | 21.8 (9.5) |  |
| III |  |  |  | 51 (22.5) | 52 (22.9) |  | 51.4 (22.7) | 51.4 (22.4) |  |
| IV |  |  |  | 69 (30.4) | 68 (30.0) |  | 68.1 (30.1) | 67.1 (29.3) |  |
| Unstaged |  |  |  | 16 (7.0) | 18 (7.9) |  | 15.6 (6.9) | 16.6 (7.3) |  |
| Pathological grade, n (%) | 0.287 | 0.171 | 0.09 |  |  | 0.683 |  |  | 1.000 |
| Grade I |  |  |  | 9 (4.0) | 11 (4.8) |  | 11.4 (5.0) | 11.8 (5.1) |  |
| Grade II |  |  |  | 71 (31.3) | 66 (29.1) |  | 65.3 (28.8) | 68.3 (29.8) |  |
| Grade III |  |  |  | 53 (23.3) | 46 (20.3) |  | 51.4 (22.7) | 49.6 (21.7) |  |
| Grade IV |  |  |  | 0 (0.0) | 1 (0.4) |  | 1.2 (0.5) | 1.0 (0.4) |  |
| Unstaged |  |  |  | 94 (41.4) | 103 (45.4) |  | 97.1 (42.9) | 98.5 (43.0) |  |
| Surgery record, yes, n (%) | 0.307 | <0.001 | 0.02 | 84 (37.0) | 84 (37.0) | 1.000 | 85.3 (38.3) | 88.7 (39.4) | 0.787 |
| Radiation record, yes, n (%) | 0.075 | 0.013 | 0.08 | 30 (13.2) | 29 (12.7) | 1.000 | 35.0 (15.7) | 28.9 (12.9) | 0.330 |
| Chemotherapy record, yes, n (%) | 0.100 | 0.082 | 0.11 | 100 (44.1) | 91 (40.8) | 0.442 | 103.2 (46.3) | 92.5 (41.1) | 0.213 |

**Abbreviation:** PSM: propensity score matching; IPW: inverse probability of treatment weighting; AFP, serum alpha fetoprotein; AJCC, American Joint Committee on cancer
