# Supplementary material for: Impact of liver fibrosis score on prognosis after common therapies for intrahepatic cholangiocarcinoma: a propensity score matching analysis
Source: BMC Cancer. 2020 Jun 15;20:556. doi: 10.1186/s12885-020-07051-5 (PMC7296657; doi:10.1186/s12885-020-07051-5)
Supplement: Supplementary file 2 — Additional file 2: Supplemental Figure 1. Subgroup survival analyses for the prognosis of ICC patients with fibrosis scores. [file 12885_2020_7051_MOESM2_ESM.pdf]

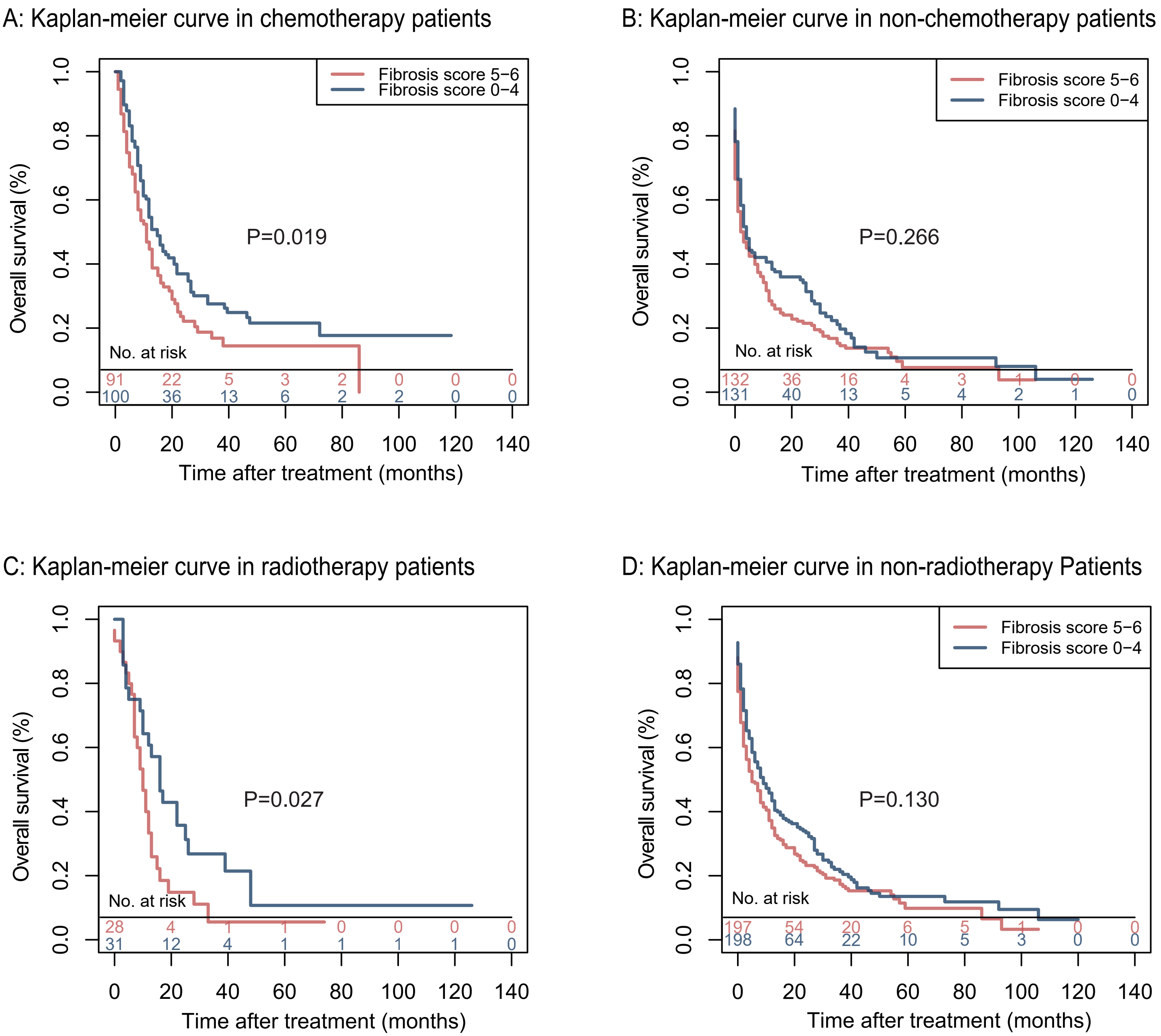
A: Kaplan-meier curve in chemotherapy patients
Fibrosis score 5−6
Fibrosis score 0−4
P=0.019
No. at risk
91 22 5 3 2 0 0 0
100 36 13 6 2 2 0 0
Overall survival (%)
Time after treatment (months)
B: Kaplan-meier curve in non-chemotherapy patients
Fibrosis score 5−6
Fibrosis score 0−4
P=0.266
No. at risk
132 36 16 4 3 1 0 0
131 40 13 5 4 2 1 0
Overall survival (%)
Time after treatment (months)
C: Kaplan-meier curve in radiotherapy patients
P=0.027
No. at risk
28 4 1 1 0 0 0 0
31 12 4 1 1 1 1 0
Overall survival (%)
Time after treatment (months)
D: Kaplan-meier curve in non-radiotherapy Patients
Fibrosis score 5−6
Fibrosis score 0−4
P=0.130
No. at risk
197 54 20 6 5 1 0 0
198 64 22 10 5 3 0 0
Overall survival (%)
Time after treatment (months)
