# Supplementary figures and images for: Impact of liver fibrosis score on prognosis after common therapies for intrahepatic cholangiocarcinoma: a propensity score matching analysis
Source: BMC Cancer. 2020 Jun 15;20:556. doi: 10.1186/s12885-020-07051-5 (PMC7296657; doi:10.1186/s12885-020-07051-5)

A

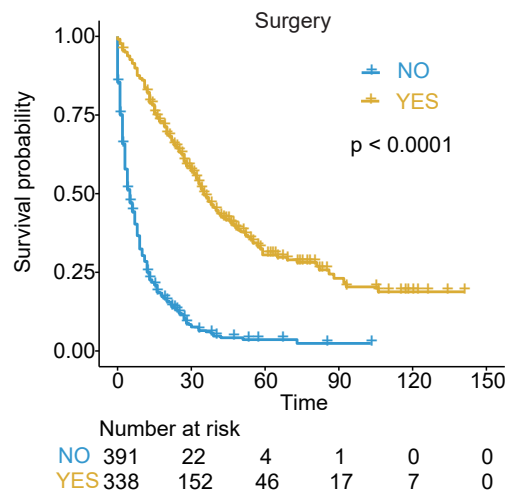

B

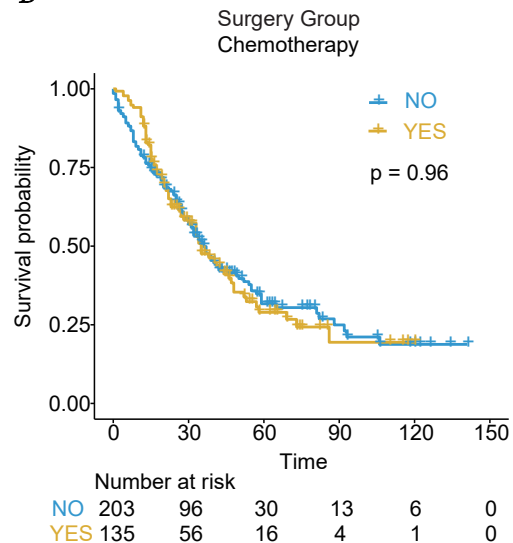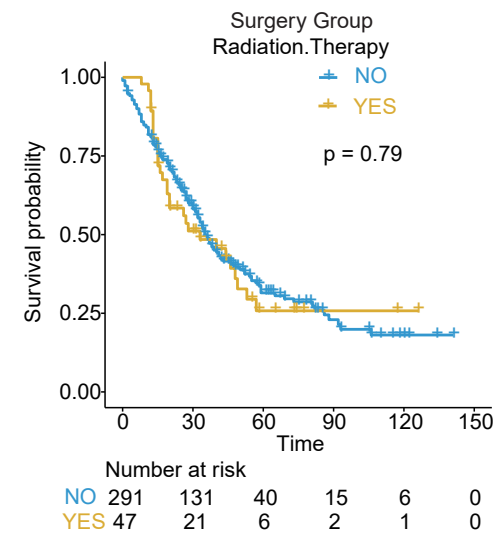

C

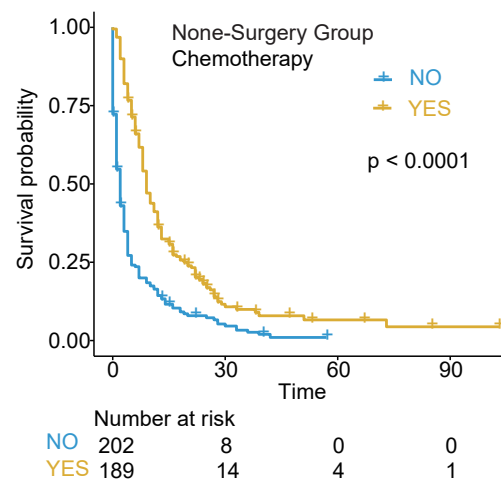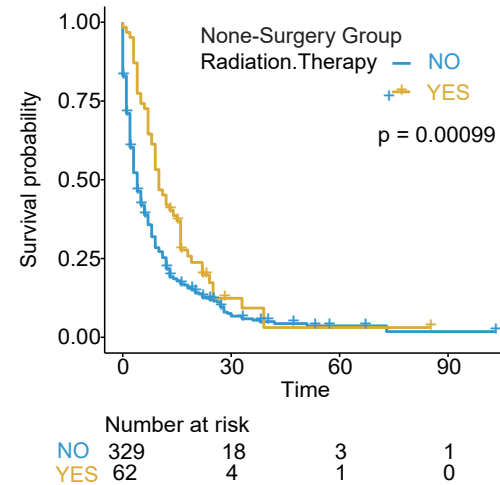

Supplement: Supplementary file 3 — Additional file 3: Supplemental Figure 2. Subgroup analysis for the prognosis of ICC patients receiving different therapies. [file 12885_2020_7051_MOESM3_ESM.pdf]
